# Effect of horizontal margin status and risk of local recurrence after endoscopic submucosal dissection for superficial esophageal cancer

**DOI:** 10.1002/jgh3.12233

**Published:** 2019-08-02

**Authors:** Hiromu Fukuda, Ryu Ishihara, Yusaku Shimamoto, Mitsuhiro Kono, Kentaro Nakagawa, Masayasu Ohmori, Kenshi Matsuno, Hiroyoshi Iwagami, Shuntaro Inoue, Taro Iwatsubo, Hiroko Nakahira, Noriko Matsuura, Satoki Shichijo, Akira Maekawa, Takashi Kanesaka, Yoji Takeuchi, Koji Higashino, Noriya Uedo, Masanori Kitamura, Shinichi Nakatsuka

**Affiliations:** ^1^ Department of Gastrointestinal Oncology Osaka International Cancer Institute Osaka Japan; ^2^ Department of Pathology Osaka International Cancer Institute Osaka Japan

**Keywords:** endoscopic submucosal dissection, esophageal squamous cell carcinoma, horizontal margin status, local recurrence

## Abstract

**Background and Aim:**

Endoscopic submucosal dissection (ESD) sometimes results in *en* bloc resection with a positive or inconclusive horizontal margin (HM1 or HMX, respectively) on histological evaluation. The specific risk for such situations is unclear. We therefore investigated the outcome of ESD with HM1 or HMX.

**Methods:**

This single‐center retrospective study was performed at Osaka International Cancer Institute. A total of 886 esophageal squamous cell carcinoma lesions in 749 patients treated from April 2005 to June 2015 were evaluated according to the following inclusion criteria: *en* bloc resection with no residual lesion, HM1 or HMX status, no prior treatment, and no additional treatment. We classified HM1 and HMX into type A, in which cancer was exposed on the HM, and type B, in which the HM status was unclear because of mechanical or thermal damage. We further classified type B according to the distance between the cancer and the edge of the specimen: type B1, <1 mm and type B2, ≥1 mm.

**Results:**

The resection margin was judged as HM1 or HMX in 5.0% (39/767; 95% confidence interval, 3.5–6.6%) of the *en* bloc resected specimens. Of 39 lesions, 30 fulfilled the inclusion criteria. Local recurrence developed in 8 of 30 lesions (26.7%). The local recurrence rates for types A, B1, and B2 were 40% (6/15 lesions), 28.5% (2/7 lesions), and 0.0% (0/8 lesions), respectively.

**Conclusions:**

Although a statistical analysis was not conducted because of the limited events, the pathological HM status may be a useful predictor of local recurrence.

## Graphical abstract

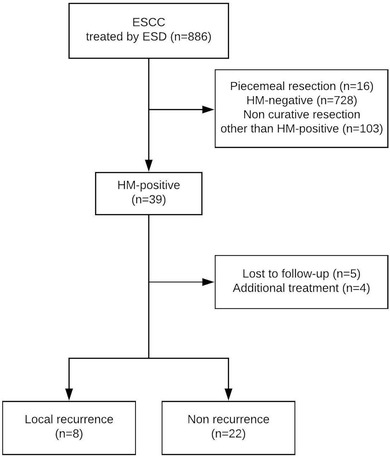

This study is the first to investigate the association between the horizontal margin (HM) status and local recurrence in patients with esophageal squamous cell carcinoma, and we suggest that the pathological HM status may be a useful predictor of local recurrence.

## Introduction

Esophageal cancer is the sixth most common cause of cancer‐related mortality worldwide.^1^ Although the incidence of esophageal adenocarcinoma is rapidly increasing in Europe and North America, squamous cell carcinoma (SCC) remains the most common tumor type.^1^ En bloc esophagectomy with regional lymph node dissection has been considered the standard therapy. Nevertheless, *en* bloc esophagectomy is associated with high mortality (4–19%),^2^ high postoperative morbidity (20–47%),^3^ and a low postoperative quality of life.^4^ Endoscopic resection has been used as a minimally invasive treatment for early‐stage esophageal cancer.^5^, ^6^ Endoscopic mucosal resection (EMR) was initially developed and applied to the treatment of early‐stage gastrointestinal cancer.^7^ Despite its efficacy, this method is sometimes associated with local recurrence or inconclusive histological results, especially when lesions of >20 mm are resected in a piecemeal manner.^8^, ^9^ The presence or absence of cancer cells at the margin of the resected specimens and the performance of piecemeal resection are reported to be predictors of local recurrence after EMR.^9^, ^10^, ^11^, ^12^

Recently, endoscopic submucosal dissection (ESD)^5^ has been increasingly used for the treatment of esophageal SCC (ESCC). ESD allows complete resection, defined as *en* bloc resection with a cancer‐free margin, in >90% of cases; this is a much higher rate than that of EMR.^9^, ^13^, ^14^ However, ESD sometimes results in incomplete resection with a horizontal margin (HM)‐positive status, especially when circumferential spread of the lesion is large or the lesion is located in a difficult location to remove. The impact of an HM‐positive status in ESD‐resected specimens has been investigated in various organs. Sekiguchi *et al*.^15^ evaluated 3784 early gastric cancers treated by ESD. Local recurrence was found in 10 of 77 HM‐positive lesions. The risk factor for local recurrence was cancer exposed on the HM for ≥6 mm. Lee *et al* 16 evaluated 527 colorectal lesions treated by ESD. They concluded that the local recurrence rate was not higher in HM‐positive cases than in R0 resection cases if the colorectal epithelial neoplasia was removed in an *en* bloc manner using ESD.

Wen *et al*.^17^ evaluated 145 esophageal cancers treated by ESD. They showed that the lesion size and cancer invasion depth were risk factors of an HM‐positive status. However, they did not show an association between an HM‐positive status and the risk of local recurrence. More detailed investigation to identify the risk factors of local recurrence in patients with an HM‐positive status would provide important information for the treatment strategy in such patients. We therefore conducted a retrospective study to identify risk factors for local recurrence in HM‐positive patients after ESD for esophageal cancers.

## Methods

### *Patients*

This single‐center retrospective study was performed at Osaka International Cancer Institute. The records for all patients who underwent ESD from April 2005 to June 2015 were extracted from the database and reviewed. The inclusion criteria were as follows: *en* bloc resection with no residual lesion on endoscopic evaluation, HM‐positive or HM‐inconclusive status on histological evaluation, no prior treatment with chemotherapy or radiation, and no additional treatment immediately after ESD.

### *ESD procedure*

ESD was performed with a conventional single‐channel endoscope (Q260J; Olympus, Tokyo, Japan) with the patient under intravenous sedation using midazolam (Dormicum; Astellas Pharma, Tokyo, Japan) and pentazocine hydrochloride (Pentazin; Sankyo Pharmaceuticals, Tokyo, Japan) or pethidine hydrochloride (Pethidine; Takeda Pharmaceuticals, Tokyo, Japan). Endoscopic knives, such as a hook knife, flush knife, or mucosectome, and injecting solution, such as 10% glycerin solution (Glycereb; Terumo, Tokyo, Japan) or 0.4% hyaluronate sodium solution (MucoUp; Boston Scientific, Tokyo, Japan), were selected based on the endoscopist's preference. The ESD process comprised four steps. First, circumferential marking dots were made 2–3 mm outside the margins of the lesion. Second, the solution was injected into the submucosa to elevate the lesion. Third, the mucosa was incised immediately outside the marking dots using the Endo Cut mode of an electrosurgical generator (VIO 300D; Erbe Elektromedizin GmbH, Tubingen, Germany). Finally, submucosal dissection was performed using the Endo Cut mode or Force Coagulation mode. In lesions spreading more than three‐fourths of the whole circumference of the esophagus, marking dots were made near the lesion, and the mucosal incision was performed closer to the marking dots to prevent esophageal stricture after ESD. The procedure time was measured from the start of resection to the completion of submucosal dissection.

### *Histological evaluation*

**Figure 1:**
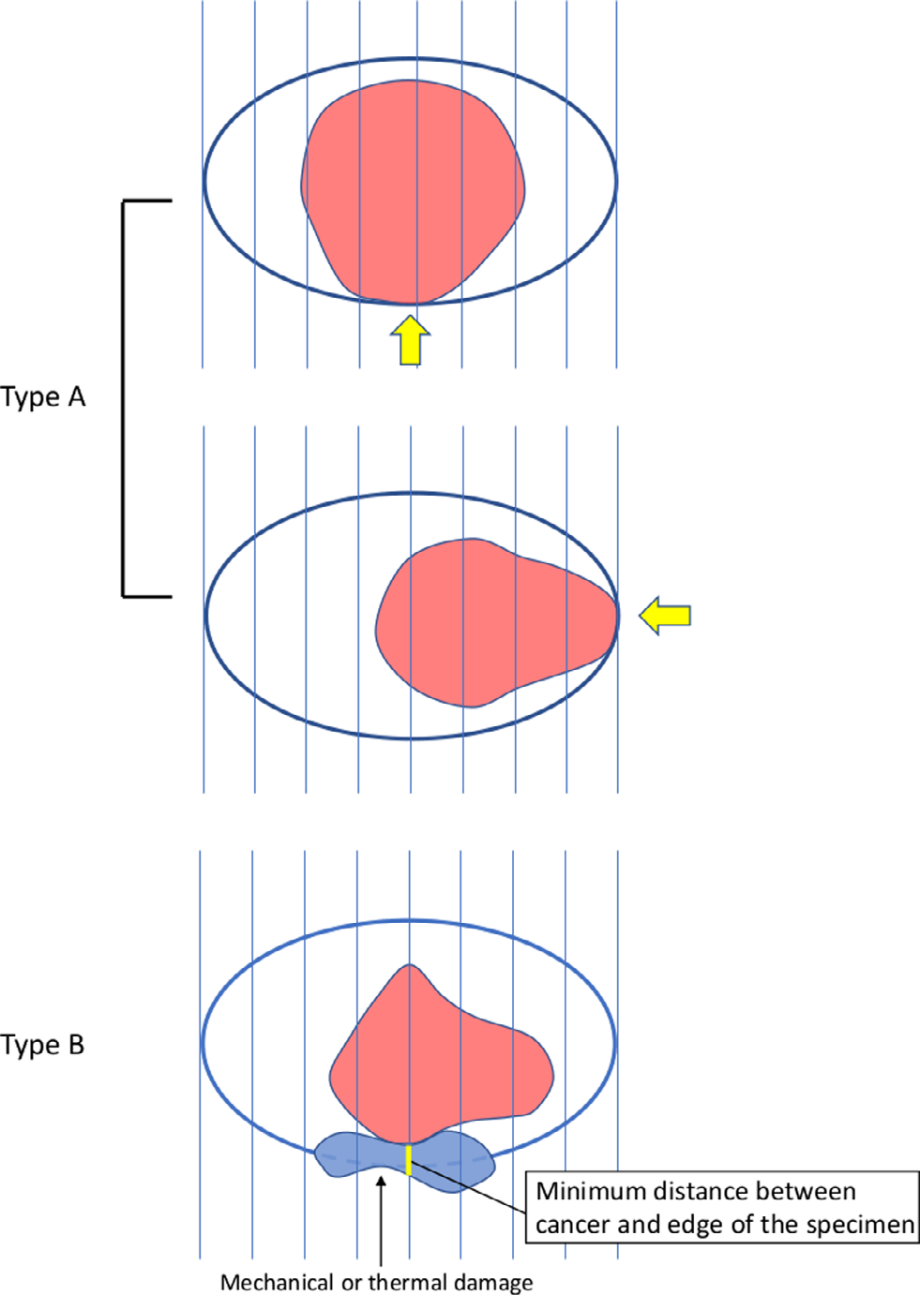
Type of horizontal margin status. Type A was defined as cancer exposed on the horizontal resection margin and type B as an unclear horizontal margin status due to mechanical or thermal damage. The minimum distance between the cancer and the edge of the specimen was evaluated (yellow line). Type B was further classified into types B1 and B2 (<1‐mm and ≥1‐mm distance between the cancer and the edge of the specimen, respectively).

The resected specimens were fixed in formalin solution, cut into 2 mm‐thick sections, and embedded in paraffin. The cross‐sectional surface subjected to hematoxylin and eosin staining and the edge of the specimen were assessed as the outmost sections. Histological assessment of the resected specimens was performed according to the Japanese Classification of Esophageal Carcinoma.^18^ Histologically curative resection was defined as *en* bloc tumor resection with a tumor invasion depth of epithelium (EP) to SM1 (submucosal invasion of ≤200 μm), no lymphovascular involvement, and tumor‐free margins. The HM was described as HM‐positive if cancer was exposed on the HM of the specimen and as HM‐inconclusive if the HM status was unclear because of mechanical or thermal damage. We further reviewed HM‐positive and HM‐inconclusive cases and classified them into two HM types: type A, in which cancer was exposed on the HM, and type B, in which the HM status was unclear because of mechanical or thermal damage. We further classified type B according to the distance between the cancer and the edge of the specimen: type B1, <1 mm and type B2, ≥1 mm (Fig. 1).

### *Follow‐up*

**Figure 2:**
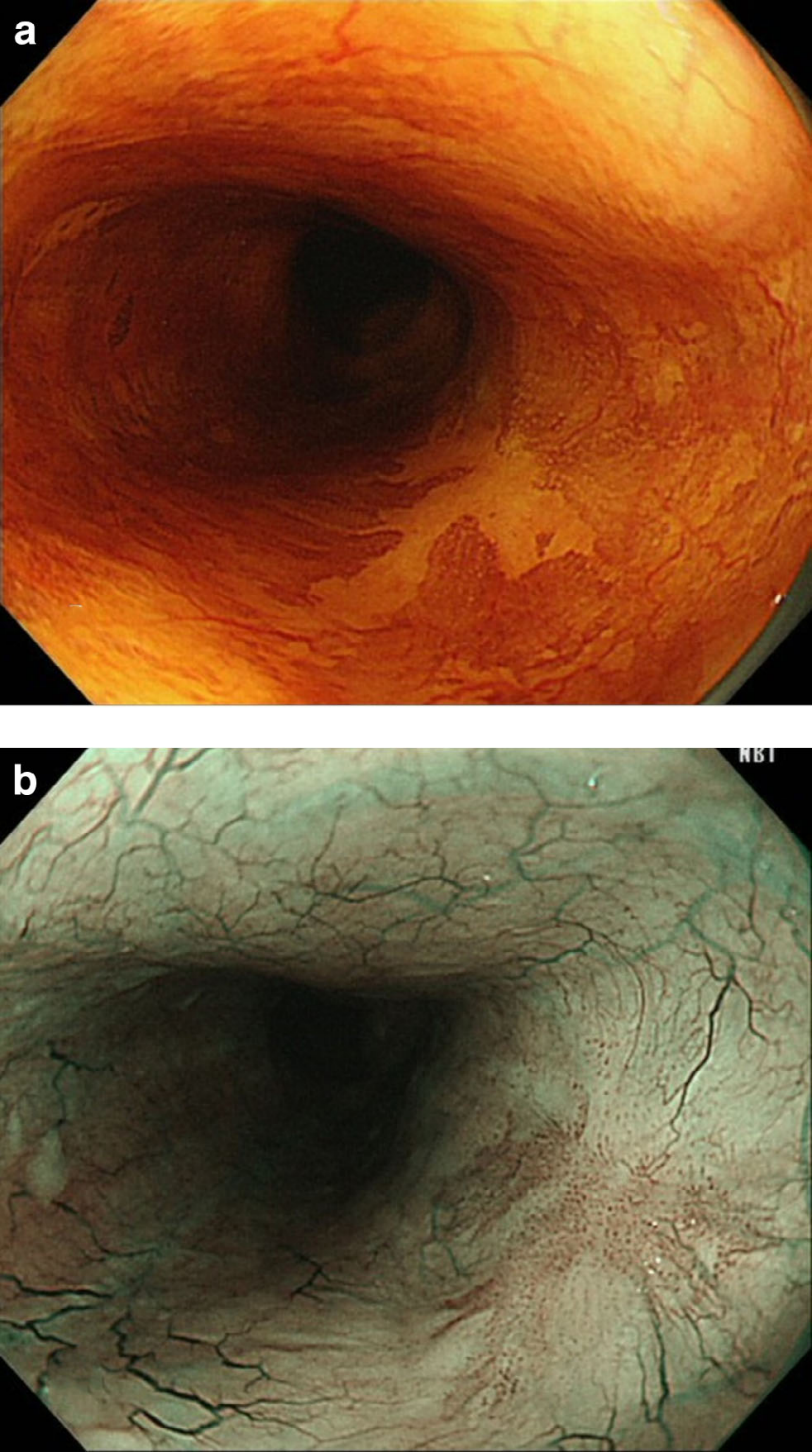
Definition of local recurrence after endoscopic submucosal dissection. (a) A diagnosis of local recurrence of squamous cell carcinoma was made based on the endoscopic finding of chromoendoscopy with iodine staining. (b) Brownish neoplastic mucosa was present beside the scar after endoscopic submucosal dissection on endoscopy with narrow‐band imaging.

After ESD, the patients were included in a follow‐up program. Upper gastrointestinal endoscopy was conducted at 2–3 months, 9–12 months, and annually thereafter. The median follow‐up period was 65 months (range, 30–149 months). In HM‐positive or HM‐inconclusive cases, additional endoscopic examinations were conducted based on the judgments of the attending physician. At least once a year, computed tomography of the neck, chest, and abdomen was performed for patients with cancer invading the muscularis mucosa or deeper level to detect lymph node or distant metastasis. Local recurrence was diagnosed when cancer was detected adjacent to the ESD scar during follow‐up (Fig. 2). The patients' follow‐up data were obtained from the medical charts.

### *Statistical analysis*

Quantitative data are shown as mean (SD) or median (range) as appropriate and were compared using Student's t test. The χ^2^ test was used for comparisons of categorical variables. Differences were considered significant at values of *P* < 0.05. All analyses were performed on a personal computer using EZR software packages; EZR version 1.27 (Saitama Medical Center, JichiMedical University, Japan).

## Results

**Figure 3:**
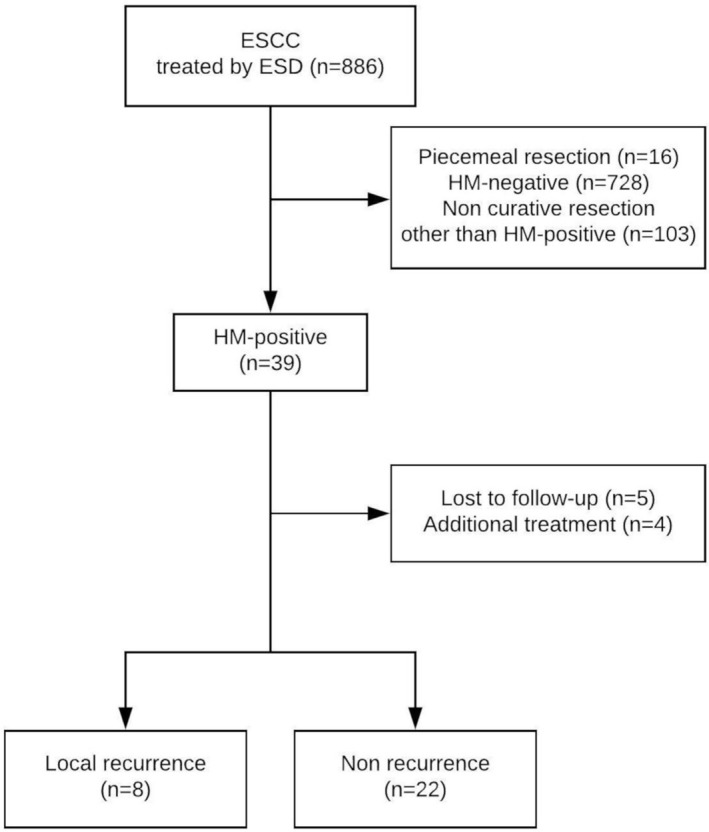
Patient selection and occurrence of local recurrence. The study included 886 esophageal cancers treated by ESD from April 2005 to June 2015. ESCC, esophageal squamous cell carcinoma; ESD, endoscopic submucosal dissection; HM, horizontal margin.

From April 2005 to June 2015, a total of 886 ESCC lesions in 749 patients were treated by ESD. Of these lesions, 39 (5.0%) were resected *en* bloc with an HM‐positive or HM‐inconclusive status (Fig. 3). Of these 39 lesions, HM was positive because of diagnosis‐related factors in 7 cases, resection‐related factors in 16 cases, and unknown factors in 16 cases. Seven cases with HM positivity based on diagnosis‐related factors had an unclear margin because of multiple Lugol‐voiding lesions. Twelve cases with HM positivity based on resection‐related factors had lesions spreading more than three‐fourths of the whole circumference of the esophagus and were resected immediately outside of the lesion to prevent stricture. In four cases with HM positivity based on resection‐related factors, ESD was difficult because the lesions were located near the surgical anastomosis. A tumor location in the cervical esophagus (*P* < 0.01) and a tumor size of ≥20 mm in diameter (*P* < 0.01) were significantly associated with an HM‐positive or HM‐inconclusive status (Table 1).

**Table 1:** Features of the lesions

	HM‐negative (*n* = 728)	HM‐positive (*n* = 39)	*P* value
Age, years	67 (39–87)	68 (52–79)	0.67
Gender			0.30
Male	616	35	
Female	136	4	
Location			<0.01
Ce	24	4	
Ut	86	10	
Mt	388	19	
Lt	175	4	
Ae	52	2	
Tumor size, mm			<0.01
≤20	328	8	
≥21	400	31	
Macroscopic type			<0.01
0–IIa	57	5	
0–IIb	44	31	
0–IIc	625	3	
Depth			0.16
EP	174	5	
LPM	451	30	
MM	102	4	

Data are presented as median (range) or *n*.

Ae, abdominal esophagus; Ce, cervical esophagus; EP, epithelium; HM, horizontal margin; LPM, lamina propria mucosa; Lt, lower thoracic esophagus; MM, muscularis mucosa; Mt, middle thoracic esophagus; Ut, upper thoracic esophagus.

After excluding 9 lesions that were lost to follow‐up, 30 lesions were included in the analysis to identify risk factors for local recurrence (Fig. 3). Local recurrence developed in 8 of 30 lesions (26.7%) during a median follow‐up period of 22 months (range, 7–35 months) (Table 2). Of these eight recurrences, seven occurred in men, and one occurred in a woman, and the patients' median age was 68 years (range, 53–78 years). Three lesions were ≥ 30 mm in diameter, and three lesions were more than three‐fourths of the whole circumference. Of the eight local recurrences, six were precisely detected and demarcated by narrow‐band imaging, while the other two were difficult to demarcate by narrow‐band imaging. We failed to identify any risk factors, probably because of the limited number of local recurrences (Table 2).

**Table 2:** Comparison of patients with and without local recurrence among those with a positive horizontal margin after endoscopic submucosal dissection

	Local recurrence (*n* = 8)	No recurrence (*n* = 22)	*P* value
Age, years	68 (53–78)	69 (52–79)	0.64
Gender			
Male	7	20	1
Female	1	2	
Location			
Ce	1	4	0.34
Ut	2	5	
Mt	4	9	
Lt	0	4	
Ae	1	0	
Tumor size, mm			
≤29	5	9	0.52
≥30	3	13	
Proportion of circumferential extension			
<1/2	2	5	0.92
1/2–3/4	3	7	
>3/4	3	10	
Macroscopic type			
0–IIa	1	3	0.21
0–IIb	0	4	
0–IIc	7	15	
Depth			
EP	0	4	0.36
LPM	7	14	
MM	1	4	
Procedure time, min	110 (91.4)	101 (47.3)	0.74
Multiple Lugol‐voiding lesions			
+	5	9	0.52
−	3	13	

Data are presented as median (range), *n*, or mean (SD).

Ae, abdominal esophagus; Ce, cervical esophagus; EP, epithelium; LPM, lamina propria mucosa; Lt, lower thoracic esophagus; MM, muscularis mucosa; Mt, middle thoracic esophagus; Ut, upper thoracic esophagus.

In the analysis of HM types, 6 of 15 type A lesions (40%) and 2 of 7 type B1 lesions (28.5%) locally recurred; however, none of the 8 type B2 lesions recurred during the follow‐up period (Table 3). The characteristics of each of these eight locally recurrent tumors are shown in Table 4. Six lesions were treated by additional endoscopic resection, and two lesions were treated by radiotherapy and chemoradiotherapy. No patients developed lymph node or distant metastasis.

**Table 3:** Local recurrence rates according to horizontal margin types

	Local recurrence rate
Type A	40.0% (6/15)
Type B1	28.5% (2/7)
Type B2	0.0% (0/8)

**Table 4:** Demographic features of eight patients with local recurrence

Case no.	Age (years)	Gender	Location	Macroscopic type	Depth	Tumor size (mm)	Proportion of circumferential extension	Procedure time (min)	Horizontal margin status	Time to recurrence (months)	Additional treatment	Recurrence after additional treatment
1	78	Female	Ut	0–IIc	LPM	48	>3/4	93	Type B1	26	RT	−
2	69	Male	Mt	0–IIc	LPM	46	>3/4	72	Type A	19	EMR	−
3	66	Male	Ce	0–IIc	LPM	8	<1/2	55	Type A	7	ESD	−
4	69	Male	Ut	0–IIc	MM	6	<1/2	32	Type A	9	ESD	−
5	66	Male	Mt	0–IIc	LPM	25	1/2–3/4	52	Type A	7	ESD	−
6	70	Male	Mt	0–IIc	LPM	23	1/2–3/4	30	Type A	25	CRT	−
7	56	Male	Mt	0–IIc	LPM	26	1/2–3/4	114	Type B1	35	EMR	+
8	53	Male	Ae	0–IIa	LPM	30	>3/4	328	Type A	35	ESD	−

Ae, abdominal esophagus; Ce, cervical esophagus; CRT, chemoradiotherapy; EMR, endoscopic mucosal resection; ESD, endoscopic submucosal dissection; LPM, lamina propria mucosa; MM, muscularis mucosa; Mt, middle thoracic esophagus; RT, radiotherapy; Ut, upper thoracic esophagus.

## Discussion

In this study, the en bloc resection rate and en bloc resection rate with a cancer‐free margin were 98.1 and 93.4%, respectively. These results are in the range of previous studies.^19^, ^20^, ^21^ ESD can theoretically achieve *en* bloc resection with an HM‐negative status in all cases. However, it may sometimes result in incomplete resection because of technical problems or strategies to avoid stricture.

Wen *et al*.^17^ reported that the macroscopic type, cancer invasion depth, and lesion size were significantly associated with positive resection margins, including the vertical margin. Among these factors, the cancer invasion depth may be related to the vertical margin, while the lesion size may be related to the HM. Investigating the vertical margin and HM independently may provide more accurate information because factors associated with a positive vertical margin and HM may differ significantly. To the best of our knowledge, this study is the first to investigate the association between the HM status and local recurrence in patients with ESCC. In this study, a tumor location in the cervical esophagus (*P* < 0.01) and a tumor size of ≥20 mm (*P* < 0.01) were significantly associated with an HM‐positive or HM‐inconclusive status. A larger lesion size and lesions located in the cervical esophagus are risk factors for stricture after ESD. A possible explanation for the higher rate of an HM‐positive and HM‐inconclusive status in these lesions may be a smaller resection margin to prevent stricture. In addition, the narrow space of the cervical esophagus may have caused difficulty in ESD and resulted in an HM‐positive or HM‐inconclusive status in some cases.

The spread of ESCC can be delineated by iodine staining. Theoretically, local complete remission can be achieved by ESD just by removing all unstained areas. In the present study, however, local recurrence occurred in 26.7% of HM‐positive or HM‐inconclusive cases even when all endoscopically recognizable areas were removed by ESD. Small invisible areas of remnant cancer after ESD may be the cause of local recurrence. Local recurrences were detected during a median follow‐up period of 22 months (range, 7–35 months). These results are substantially equivalent to the range of previous studies because >80% of cancers reportedly recur within 18 months.^9^ We failed to identify risk factors for recurrence because of the small number of cases of recurrence. However, collecting enough cases of recurrences may be quite difficult considering that we could not collect enough lesions among >800 cases of ESD.

The risk of local recurrence in patients with an HM‐positive status has been previously investigated in other organs. However, this risk has not been investigated in patients with an HM‐inconclusive status due to a burning effect or mechanical artifact. Based on the present study, substantial risk may exist if the cancer spreads <1 mm to the lateral edge with a burning effect or mechanical artifact. In contrast, no recurrence occurred if the cancer spread ≥1 mm to the lateral edge with a burning effect or mechanical artifact. This information may help to determine follow‐up schedules in patients with an HM‐inconclusive status. The most highly recommended strategy is strict surveillance of patients with type A and B1 cancer with endoscopic treatment of local recurrence. An alternative strategy would be prophylactic ablation therapy such as argon plasma coagulation.

This study is limited by its single‐center and retrospective design. A prospective study is needed to further confirm the results of the present study.

In conclusion, although a statistical analysis was not conducted because of the limited events, the pathological HM status may be a useful predictor of local recurrence. HM‐positive cases and HM‐inconclusive cases with a <1‐mm distance between the cancer and specimen edge had substantial risk of local recurrence, and strict follow‐up is recommended for these cases. In contrast, cases with an HM‐inconclusive status with a ≥1‐mm distance between the cancer and specimen edge had no recurrence.
